# Perceived Value of Using a Digital Tool to Screen for Elder Mistreatment in the Emergency Department

**DOI:** 10.1093/geroni/igaa057.631

**Published:** 2020-12-16

**Authors:** Fuad Abujarad, Thomas Gill, Michael Pantalon, Karen Jubanyik, James Dziura, Gail D’Onofrio, Esther Choo

**Affiliations:** 1 Yale University, New Haven, Connecticut, United States; 2 Oregon Health & Science University, Portland, Oregon, United States

## Abstract

A major barrier to reducing Elder Mistreatment (EM) is an inability to accurately identify victims. We conducted a qualitative study to evaluate stakeholders’ perceived value and likelihood of adopting a tablet-based digital health tool to facilitate screening and prompt self-disclosure of EM in emergency departments (ED). The interactive tool utilizes virtual coaching, interactive multimedia libraries (graphics, animations, etc.), electronic screening, and brief motivational interviewing designed to enhance identifying EM among older adults. We conducted 3 focus groups with stakeholders, including 24 adults 60+ years, 2 social workers, 2 caregivers, and 2 ED clinicians. Two focus groups included only older adults, while one included representatives of all stakeholders. The main findings include: using a female voice for the tool narrator, larger font size, more multimedia, and headphones for privacy; and making a person available during screening if assistance is needed. Stakeholders indicated that it is difficult for victims to ask for help and any type of mistreatment screening would be helpful. On a 7-point Likert scale ranging from “1=Very Comfortable” to “ 7=Very Uncomfortable”, older adults scored 2.8 on average for whether they would feel comfortable using a tablet to screen for EM. Some said digital screening would maintain privacy and anonymity. Stakeholders highlighted the need to explain community resources available to older adults once EM is disclosed, especially resources offering help to the caregiver. In summary, this qualitative study supported using tablet-based screening for EM and highlighted the need to target stigma related to EM disclosure and fear of retaliation.

